# Supplementary material for: Differential Inhibition of Target Gene Expression by Human microRNAs
Source: Cells. 2019 Jul 30;8(8):791. doi: 10.3390/cells8080791 (PMC6721455; doi:10.3390/cells8080791)
Supplement: Supplementary file 1 [file cells-08-00791-s001.pdf]

Table S1. List of the tested genes. Confirmed targets and their average R1 values are shown. R1 is defined in the main text. Genes in italic were not confirmed as targets in this study. Marked in red are previously known target genes, with reporter assay evidence, according to miRTarBase.

miR-1		miR-122		miR-124	
Gene	R1	Gene	R1	Gene	R1
MAB21L1	3.67	SFT2D1	3.58	PIM3	2.37
SAMSN1	3.20	PAPOLA	3.41	PPP1R13L	2.09
E2F5	3.37	NPEPPS	3.42	RYR3	2.41
PDE7A	3.48	PIGS	3.88	VPS35	3.50
ETS1	3.86	ZBTB4	3.54	ASPA	2.76
C6orf222	5.03	LUZP1	4.00	MIPOL1	3.83
OTX2	5.19	PLAG1	4.19	PGM2	2.44
NOTCH3	3.94	BHLHE41	3.54	SERINC2	4.23
SOX9	5.29	PDK4	4.22	SNX16	3.12
SATB2	5.57	SLC2A14	3.67	QSER1	3.51
VAMP2	4.71	TNRC6A	3.74	FOXQ1	2.11
HELZ2	4.28	CCNG1	4.32	YEATS2	2.66
MON2	3.50	POLR3D	4.40	NECAP2	2.64
NXT2	3.67	POMT2	5.58	ZFP36L1	3.89
GTF3C1	5.44	IGSF5	4.46	RFX6	3.14
SLC25A30	4.02	PKM	4.31	LAD1	3.38
RASA1	5.47	RFXAP	4.24	NUMA1	3.36
PRKCI	5.36	STOML3	4.55	HMGXB4	4.12
CCDC121	5.38	RBM47	4.34	THAP2	3.76
HMGN1	4.73	CS	4.40	CHSY1	4.68
HAND2	4.01	MAPRE1	4.25	SUCLG2	2.98
ZNF800	4.17	BCL2L2	4.73	RANBP10	5.05
NEUROD1	4.10	BIRC5	4.81	DPH3	3.63
L3MBTL4	5.06	TCP10L	4.47	LITAF	3.01
MEX3C	4.13	MBNL1	4.77	GRIA3	4.74
GNPTAB	4.65	NT5C3A	4.94	TOR3A	3.88
KAT6B	6.42	SLC9A6	4.39	HIPK3	3.11
TFAP2C	6.51	NAB2	4.62	UBE3A	3.86
WDR1	4.29	GRPR	5.11	RFX1	3.00
TGIF1	3.93	RDH12	3.83	TMEM267	4.24
GNE	6.13	CTNND2	4.82	MAN2A1	4.15
C9orf82	5.42	GALNT12	4.59	PPFIBP2	5.33
WDR78	6.23	USP8	4.50	LHX2	4.49
ANXA2	5.39	ZNF827	4.81	SVIP	2.72

SYBU	4.65	RIMS1	4.56	TMEM134	4.34
C10orf2	5.68	KIR2DS2	6.55	ZBTB11	3.38
WDR47	5.40	SMARCD1	5.06	SLC31A2	2.11
SIDT1	6.05	EPO	4.91	KLF4	3.64
GPR3	5.89	KHDRBS1	5.36	RALBP1	3.86
MBLAC2	5.44	AACS	5.09	PEA15	3.52
DCLK3	6.10	CCNYL1	4.47	SLC15A4	4.49
KLF4	4.95	DICER1	4.26	GPAT3	3.95
ACBD7	5.59	CYP4V2	6.73	EYA2	4.12
BOLL	4.95	G3BP2	4.87	PCDH8	5.53
PRLR	5.60	GRAMD3	5.34	PAQR8	5.05
WDR48	4.78	MARK1	4.82	KCNK2	4.04
GOLGB1	6.47	ADPRH	5.03	ZNF219	4.79
CBARA1	4.54	AGO1	5.40	EFNB1	4.44
IL2RA	6.49	FOXP1	5.55	EN2	4.47
KCNA5	5.41	KCNH5	5.05	SLC26A2	3.71
SIM1	6.52	UHRF1BP1	5.42	CTNS	4.93
ZNF280D	5.24	SUCLA2	5.42	RAB4A	4.15
SNAI2	4.92	ZDHHC	5.60	SLC10A7	4.10
KTN1	6.23	PHF14	4.19	ARHGAP18	4.48
KCNJ2	5.54	GCNT4	5.50	INTS6L	4.72
RFTN2	6.32	EGLN3	4.89	GCDH	4.54
PCDH17	5.97	YPEL4	4.70	TMED1	4.27
MUT	7.33	SFRS7	5.31	GNPDA2	5.83
WNT3	5.15	FAM185A	6.16	DYRK2	4.37
SYNJ1	6.63	RBP5	5.16	SESTD1	5.58
PLEKHA7	5.83	ASAH1	5.51	TSPAN6	5.70
POLR3G	5.90	FRAT1	5.97	HIPK1	4.49
PCDHB13	4.52	ZAK	5.53	GAS2	5.48
XPO6	5.75	FUT2	6.22	RBMS1	3.68
BMP3	4.83	DUSP4	5.18	NRCAM	4.49
YWHAZ	6.06	SLC25A19	4.49	LRIG1	4.32
PDGFA	5.74	F13A1	4.62	CNTN1	5.12
MYLK	5.45	NICN1	5.13	TJP2	5.52
TMEM168	6.47	TBR1	4.43	SORD	5.14
CORO1C	4.57	FUT8	5.22	KPNA3	3.18
MICU1	6.34	GNPDA1	5.33	USP1	5.57
SLAMF8	5.90	CASP6	5.93	ZWINT	4.31
YRDC	6.27	APCDD1L	5.32	RSPO3	5.35
HSBP1	5.25	ZNF614	5.81	FGFR2	4.70
LIPI	4.99	RHOA	5.50	NAP1L3	5.36
CWC15	6.70	CD320	5.33	SERTAD3	3.62

CCND2	6.27	IHH	5.22	DHRS1	4.42
TMEM55B	5.83	BRPF1	5.14	ONECUT2	4.90
TM4SF1	6.20	CTDNEP1	4.88	FLOT2	5.20
ADAM11	6.83	SP2	5.15	STT3A	5.53
RIPK4	6.52	ZC3H13	7.28	AMPD3	4.62
TPD52L3	5.89	MCM10	6.04	NAV3	4.66
MMP8	5.67	ANKRD13C	5.42	DMRT1	4.02
DHX15	6.33	PTPRF	5.52	LCLAT1	4.11
SAP30BP	6.74	FAM219A	4.96	LFNG	3.91
HSPD1	6.62	HOXC8	5.17	ALG2	5.34
BPNT1	5.19	SPECC1	6.16	RAB27A	5.31
KMT2E	5.95	NMUR2	5.52	PTPN1	5.96
PDCD10	6.40	APBA2	6.63	ADAMTS19	5.58
ATP6V1A	5.92	SLC35B2	5.58	SCNN1G	6.83
ARRDC3	5.51	SLC13A5	6.04	DDX60L	3.32
LRRC16A	6.18	PXN	4.76	SPINDOC	5.90
DAAM1	5.56	CADM2	4.95	SPOPL	3.99
ZNF827	6.08	PAICS	6.02	EYA4	5.74
SPOCK1	7.05	CSF2RB	5.04	CCDC28A	6.21
CRK	5.21	NDRG3	4.79	HDAC5	4.27
KHSRP	6.02	GALNT1	5.75	KCNJ2	3.45
PRKRIR	5.97	PTPN1	6.15	CIRBP	4.57
ARF4	5.72	PTDSS2	5.20	OSBP2	5.28
SFRP2	5.68	LOC285548	5.55	GLCE	5.27
SETBP1	5.41	SIRT2	6.21	PPP6R3	6.49
DNER	6.83	FAM107A	5.70	ACTN4	6.70
VIPAR	6.88	FOXI1	6.36	DGAT2	4.80
NR1H3	5.79	BOD1L2	6.36	LMF2	3.53
EYA4	7.18	MIP	6.36	PTGFRN	5.57
FAM72B	4.82	KDM2A	6.08	IGF2R	5.99
COL4A3	5.86	TXNDC6	6.03	TEX261	6.74
ZNF281	6.28	RGAG1	6.37	KLF12	4.65
CPED1	6.26	OLR1	5.34	GALNT12	7.31
RCAN2	6.56	CSNK1G1	5.66	CDH11	6.91
MEOX2	5.00	PLA2G2F	5.15	BACE1	3.01
MAP4K3	6.07	METTL1	4.89	ZRANB2	4.71
EIF2S3	6.73	ADAM17	5.46	RTN3	7.68
TSPAN9	6.32	CTDSPL2	7.06	RALA	4.03
WDR6	6.64	PLCB2	4.73	IMPACT	4.72
RNF40	5.76	USP40	4.79	ANXA5	4.50
NUP160	6.29	UBAC2	5.19	ZNF420	4.54
NAB1	6.86	DUOX2	6.10	MEF2A	5.51

FZD7	6.23	HNRNPU	5.99	ITGA11	2.51
SPTLC3	5.66	PIK3CG	5.24	HAND2-AS1	4.66
MOSPD2	6.49	COMMD8	5.99	CTGF	5.72
SLC37A3	5.16	P4HA1	6.03	VEZF1	4.20
MKL1	6.24	DLAT	4.98	MDN1	4.94
NDEL1	6.55	TBX4	5.27	UBOX5	7.42
GCLC	4.78	NKAIN2	5.80	PGRMC2	4.26
PIAS3	6.03	VGLL2	5.02	CCDC71L	5.01
ASH2L	5.32	MAF1	5.63	INSIG2	4.42
ZBTB4	6.11	ZNF114	6.26	USP14	6.90
FZD5	5.67	RWDD4A	6.27	FAXDC2	5.19
SYN3	5.85	RUFY4	6.08	SLC25A20	4.30
ZFP91	6.17	FOXL1	6.56	C1GALT1	4.97
CREM	6.55	SLCO5A1	5.84	ANKRD13A	6.53
NETO2	5.56	C4orf50	6.64	PM20D2	6.48
GSAP	7.58	ELOVL1	6.34	RBM24	5.05
KDM2B	5.24	MRPS10	5.35	PIEZO2	5.53
PTPRN	7.19	EPM2AIP1	6.66	EPHX4	3.44
LOC285419	6.24	SOX13	4.87	EFCAB14	5.96
DGKG	6.05	RAD21	5.50	NDFIP1	5.21
FGFBP3	7.48	PTBP3	5.57	SNCAIP	5.97
SLC6A2	6.03	PCDHGA7	6.04	CLIP4	5.37
EIF4E	7.03	KAT6B	6.34	MYO1C	5.92
CA13	6.56	GPR6	7.42	SEC61A2	5.30
ANXA4	6.29	MEIS2	5.64	G3BP1	5.78
SLC25A1	6.23	AMPH	6.57	PRPF38B	5.56
RNF14	6.39	C2orf67	6.05	PARP1	6.79
HACE1	6.30	RAP2C	5.15	*DUSP15*	
MAPKBP1	6.19	CYB561	5.58	*AGFG1*	
DGKZ	6.44	GNA13	6.39	*MTF2*	
FLJ42280	5.63	GABRE	6.01	*HIVEP2*	
STARD7	7.59	UBE2J2	6.40	*TMOD1*	
BAG4	6.36	SRD5A3	6.47	*SURF4*	
SEL1L3	6.49	IPO8	5.57	*NPLOC4*	
GYG2	5.62	ZNF22	5.95	*NCKIPSD*	
PIK3CG	6.60	C1orf123	6.55	*NEUROD1*	
MPZ	6.33	ZIM3	6.34	*DNAJB12*	
MATR3	6.78	TTC31	5.25	*RAB34*	
CXCL6	6.28	DAND5	6.69	*DICER1*	
JAZF1	6.74	GPR3	6.33	*UBE2B*	
FBP1	5.87	SLC4A3	6.67	*CLDN11*	
RSBN1L	7.46	OTOP2	6.44	*CNEP1R1*	

EDNRB	7.11	BHLHE22	6.29	*PDCD6*	
FAM104B	6.01	DPYSL4	6.46	*TSHZ1*	
ZBTB7B		CA10	5.42	*FXR2*	
TPH2		*MINK1*		*NPTN*	
TEF		*DEF8*		*AP1M2*	
CCRL1		*LCORL*		*BARX2*	
PDIK1L		*ADRB3*		*LRP6*	
DIP2C		*C15orf53*		*TTC7A*	
MECR		*CDKN1B*		*PCSK6*	
DLEU2L		*USP46*		*SGK1*	
RNF19A		*ZNF514*		*EYA1*	
GAPT		*RNF165*		*AP3M1*	
GNPNAT1		*CTCF*		*RB1CC1*	
NCL		*DDX20*		*PDCD10*	
TOR1AIP1		*DR1*		*LPCAT3*	
POF1B		*C1QTNF7*		*PSMD5*	
THAP5		*BCAT2*		*SGO2*	
ANP32B		*SLC2A3*		*SCUBE3*	
DOK3		*LLPH*		*TMEM50B*	
WNK3		*TIAL1*		*FAM81A*	
GADL1		*HAND2*		*RASGEF1A*	
TBP		*LMO3*		*OVOL2*	
VPS45		*NOL4*		*TACC2*	
TSPYL4		*BDNF*		*SPRED1*	
FAM72A		*VAV3*		*SYNCRIP*	
YWHAQ		*TMEM181*		*C10orf 12*	
NADK		*MYO1C*		*ZNF687*	
SNAP25		*DGKD*		*FZD8*	
ASRGL1		*HERC6*		*GDAP1L1*	
CCDC46		*NFAT5*		*SGPP1*	
GSTCD		*PROX2*		*CHST1*	
COPS2		*XPO6*		*DIAPH1*	
SRC		*IL1RN*		*SEMA6C*	
ATG13		*GTSE1*		*TXNDC5*	
HSPA4				*MARCH*	
MED1				*ZNF706*	

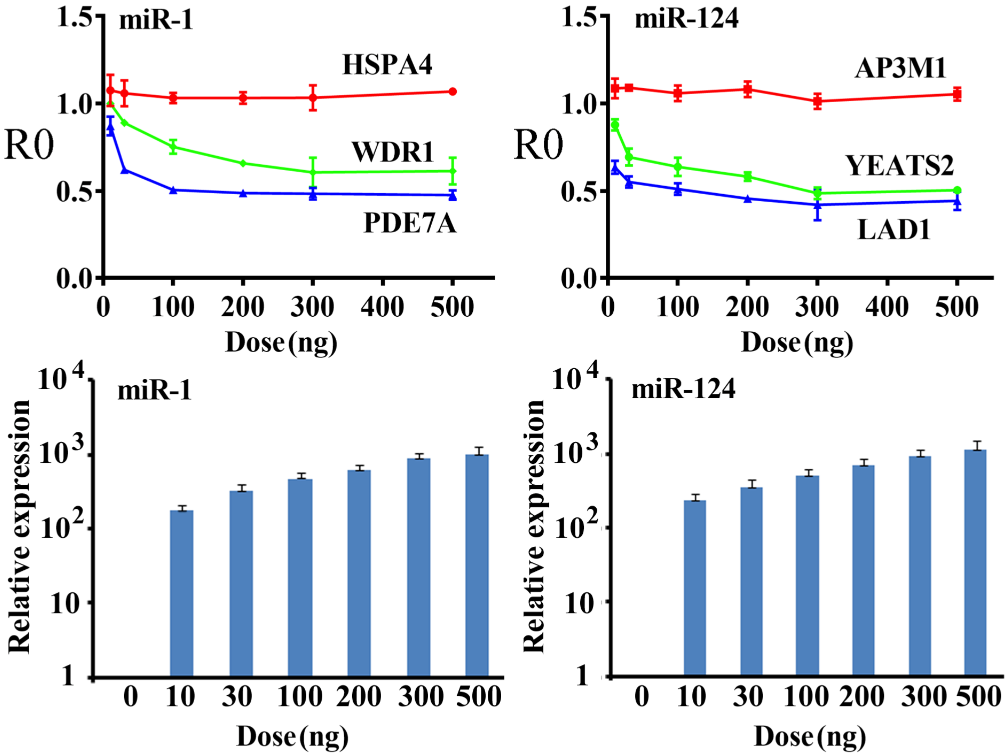

Figure S1. miRNA dose-dependent inhibition of target gene expression. 239T cells in 24-well plates were co-transfected with different amounts of miR-1 or miR-124 expressing plasmid and the indicated reporter constructs. The x-axes show the amounts of transfected miRNA-expressing plasmids. Top panels: R0 values were computed following luciferase assays, as defined in the main text, and the averages and standard deviations shown in the y-axes. Bottom panels: relative miR-1 and miR-124 expression levels in cells determined by quantitative PCR, with averages and standard deviations shown in the y-axes (expression in non-transfected cells set as 1). Relative miRNA expression was determined as described in Zhang, X.; Graves, P. R.; Zeng, Y. Stable Argonaute2 overexpression differentially regulates microRNA production. *Biochim. Biophys. Acta* **2009** *1789*, 153-159.

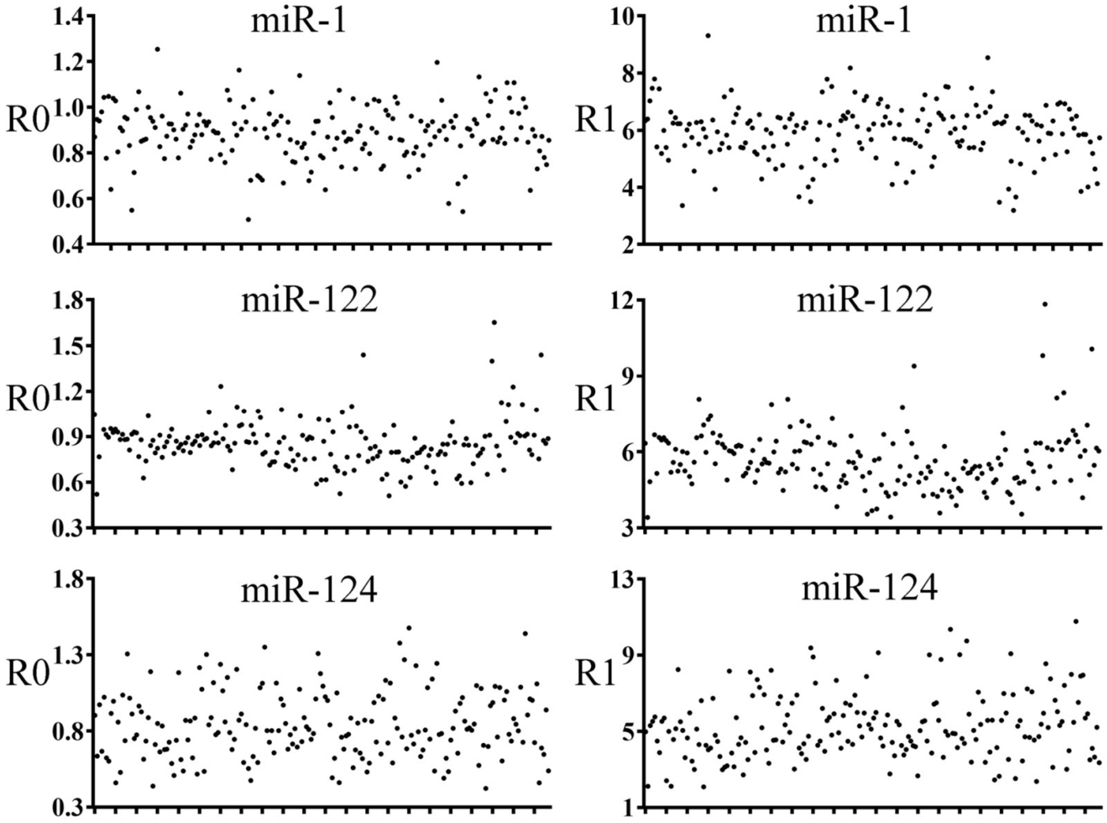

Figure S2. Averages of the R0 and R1 values (y-axes) for all the miR-1, miR-122, and miR-124 predicted targets. Dots represent individual genes along the x-axes.

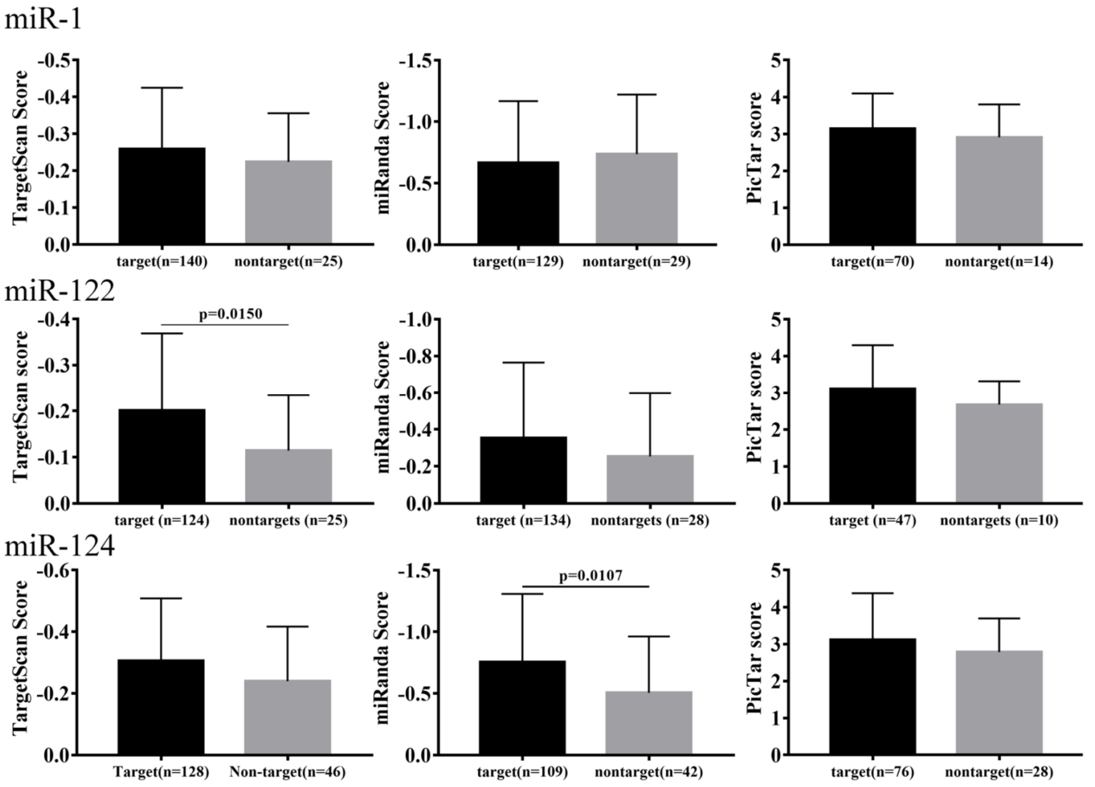

Figure S3. Comparisons of the ranking scores by TargetScan, miRanda, and PicTar of the predicted targets that were confirmed and not confirmed in the reporter assays.

Averages and standard deviations are shown, with numbers in parentheses the sample size.

When significant (< 0.05), p-values are indicated.

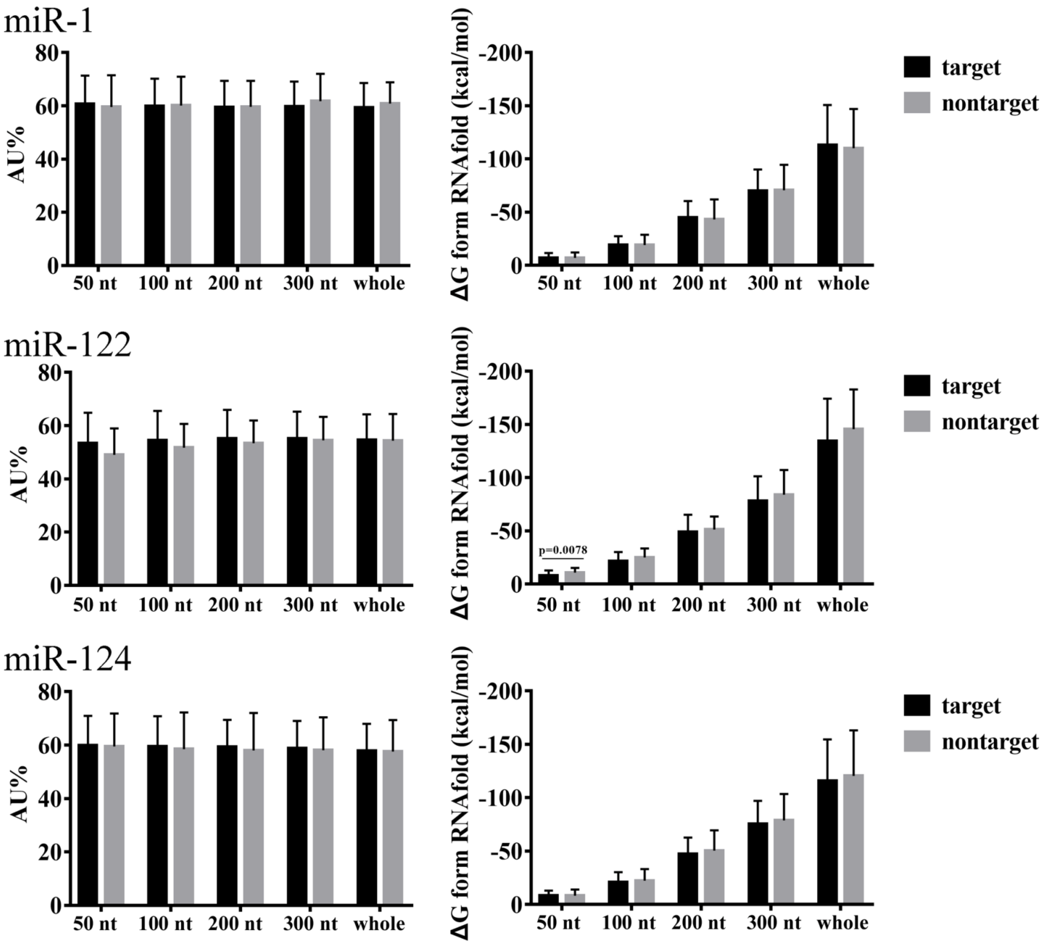

Figure S4. Comparisons of the AU% and ΔG of predicted secondary structures of the confirmed targets and unconfirmed targets. Averages and standard deviations are shown. A significant p value (< 0.05) is also shown. See Table 2 and **Figure S3** for information about labeling.

	TargetScan conserved sites			TargetScan poorly conserved sites		
	selected	confirmed	ratio	selected	confirmed	ratio
miR-1	99	87	87.9%	66	53	80.3%
miR-122	62	57	91.9%	87	67	77%
miR-124	153	110	71.9%	21	18	85.7%

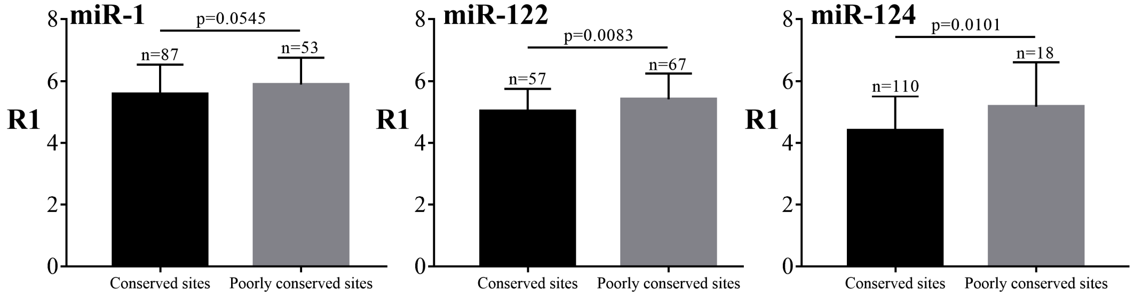

Figure S5. Comparisons of TargetScan 7.0-predicted targets with conserved sites and targets with poorly conserved sites. Top panel: Numbers of genes that were predicted by TargetScan, confirmed targets, and the ratios for miR-1, miR-122, and miR-124. Bottom panel: R1 values (y-axes) of confirmed targets with conserved sites and poorly conserved sites. Averages and standard deviations are shown. Numbers of the confirmed genes (n) and p values are also indicated.

	mirDIP	confirmed	ratio
miR-1	139	120	86.3%
miR-122	100	83	83%
miR-124	177	129	72.9%

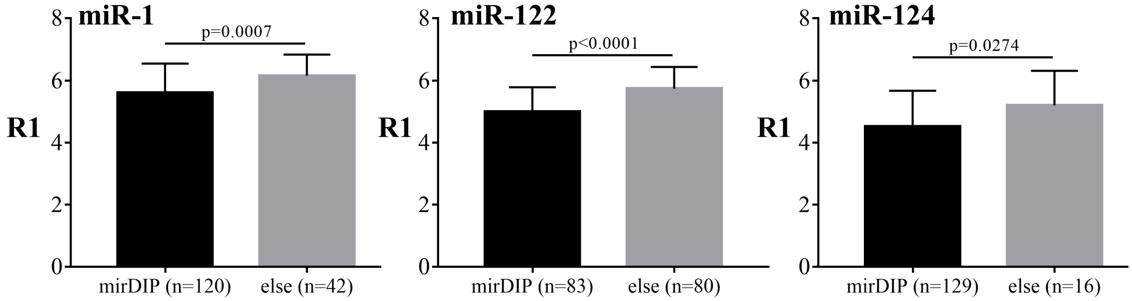

Figure S6. Targets predicted by mirDIP 4.1. Top table: Numbers of genes that were predicted by mirDIP, confirmed targets, and the ratios for miR-1, miR-122, and miR-124. Bottom panel: R1 values (y-axes) of confirmed targets predicted by mirDIP and those of the not predicted (“else”). Averages and standard deviations are shown. Numbers of the confirmed genes (n) and p values are also indicated.

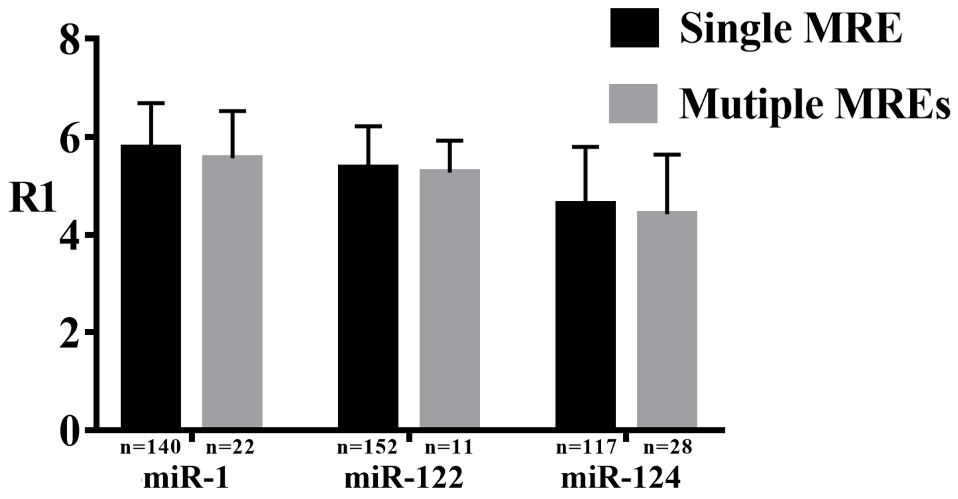

Figure S7. Comparisons of confirmed miR-1, miR-122, and miR-124 targets containing a single MRE and two or more MREs. Y-axis shows the R1 values with averages and standard deviations, sample size indicated below.
